# Identification of Key Candidate Genes and Pathways of *Candida albicans*-Infected Human Umbilical Vein Endothelial Cells and Drug Screening

**DOI:** 10.1007/s12088-019-00847-5

**Published:** 2019-12-13

**Authors:** Wei Jiang, Ping Liu, Jianlei Zhang, Wenjie Yang

**Affiliations:** 1 Department of Infectious Diseases, Tianjin First Center Hospital, No. 24 Fukang Road, Nankai District, Tianjin, 300192 China; 2 Laboratory of Microbiology of Tianjin First Center Hospital, Tianjin, China

**Keywords:** Bioinformatics analysis, *Candida albicans*, HUVECs, MYC

## Abstract

*Candida albicans* is a common opportunistic pathogen that can cause serious infection by blood transmission. *C. albicans* enters the blood circulation and adheres to the endothelial cells of the vascular wall. However, the detailed mechanism of the effect of *C. albicans* on the endothelial cells remains unclear. In this study, the microarray expression profile of human umbilical vein endothelial cells exposed to *C. albicans* was analyzed. The 191 up-regulated genes were enriched in TNF, T cell receptor, and NF-kappa B signaling pathways. The 71 down-regulated genes were enriched in pyruvate metabolic, purine nucleotide metabolic, purine nucleotide biosynthetic, and humoral immune response processes. Gene set enrichment analysis showed that apoptosis, oxidative phosphorylation, IL6/JAK/STAT3 signaling pathways were enriched. Moreover, two hub genes with a high degree of connectivity, namely, MYC and IL6, were selected. Molecular screening of traditional Chinese medicine libraries was performed on the basis of the structure of MYC protein. The okanin had the highest docking score. MYC might be used as molecular targets for treatment. In addition, okanin may inhibit the infection of *C. albicans*. Thus, MYC can be subjected to further research.

**Electronic supplementary material:**

The online version of this article (10.1007/s12088-019-00847-5) contains supplementary material, which is available to authorized users.

## Introduction

*Candida albicans* is a polymorphic yeast and one of the most important pathogens that causes iatrogenic infections in immunodeficient populations [1]. In general, it is a common parasite of humans that can be found in the oropharynx, gastrointestinal tract, and vaginal mucosa and does not cause human diseases. However, when the host’s local micro-environment is dysregulated, or the mucosal barrier is impaired, *C. albicans* can cause mucosal infections, thereby causing diseases such as thrush, fungal vaginitis, and rash [2, 3]. In some cases, *C. albicans* can invade the mucosal epithelium and vascular endothelium, thereby causing disseminated infections in susceptible populations [4]. In the pathology of the abovementioned diseases, *C. albicans* invades the host’s non-phagocytic cells, such as endothelial cells, and plays a vital role in the early stages of the disease [5]. Currently, no mechanism study has been conducted on the effects of *C. albicans* on endothelial cells.

High-throughput virtual screening revealed that okanin has the highest docking score with MYC [6, 7]. Okanin is a chalcone compound found in the genus *Bidens *[8]. Pharmacological studies have shown that okanin has many effects, such as lowering blood sugar, lowering blood pressure, lowering blood fat, and resisting oxidation [9]. Although the anti-inflammatory activity of okanin has been studied [10], the anti-*C. albicans* infection activity of okanin has not been investigated yet.

In the present study, differentially expressed genes (DEGs) of endothelial cells after *C. albicans* infection were analyzed by performing bioinformatics analysis. The key functions and pathways of endothelial cells after *C. albicans* infection were also identified. By conducting the topological analysis, we found and further screened the key hub genes in the affected endothelial cell lesions to obtain okanin. The candidate molecule for traditional Chinese medicine (TCM) may inhibit *C. albicans* infection.

## Materials and Methods

### RNA Sequence Analysis

The RNA expression profiling of GSE7355 downloaded from the GEO database was used in this study. Four independent experiments compared the expression profiles of untreated HUVEC monolayer and of *C. albicans*-infected HUVEC monolayer. The data quality was assessed by calculating residuals.sign, residuals, weight, relative log expression (RLE), normalized unscaled standard errors (NUSE), and RNA degradation. We used the R packages of pheatmap and limma to analyze the difference in RNA expression profiling among the eight groups. We set the |log2fold change| ≥ 0.6 of the cutoff limit for DEGs.

### Gene Set Enrichment Analysis (GSEA)

GSEA was conducted using the GSEA software [11].

### Omics Analysis

The DEGs were further analyzed using the gene ontology (GO), KEGG pathway, and protein–protein interaction (PPI) analyses [12]. The GO and pathway analysis were conducted using the website of Metascape (http://metascape.org). The enriched pathways or functions were then drawn into bubble maps with the R package of ggplot2. PPI annotation of the DEGs was retrieved from the STRING database. The PPI network was visualized using the Cytoscape software. Then, the Cytoscape apps of CentiScape and MCODE were used for detecting densely connected regions [13, 14].

### TCM Database and Protein Preparation

A total of 32,364 TCM molecules were obtained from the TCM database (http://tcm.cmu.edu.tw/) [15]. All the TCM molecules were refined by removing the counterions and salts and adding hydrogen atoms. Then, we performed energy minimization using Schrodinger software [16, 17]. For protein preparation, the crystal structure of MYC was downloaded from the protein data bank (http://www.rcsb.org/, PDBID: 1NKP) [18]. The protein structure was refined by removing crystalline water and ions. Then, we added hydrogen atoms and performed energy minimization of the protein structure. We selected high-throughput virtual screening model of Schrodinger to perform molecular docking. Glide XP (extra precision) was used for the final 10 TCM molecules calculations [19, 20].

### Luciferase Report Assay

The HUVEC cell lines, obtained from KeyGen Biotech (Nanjing, China), were transfected with dual-reporter constructs of MYC using the transfection regents. After transfection of 24 h, cells were treated with 40 µM of okanin. After another 48 h, the culture medium was collected into a 96-well plate and the luminescence was measured using a luminometer. The fluorescence intensity reflects the transcriptional activity.

### Western Blot Assay

The okanin was added to the HUVEC cell culture at a concentration of 40 µM when the cell density reached to 80%. After incubation of 48 h, the cells were lysed and 30 µg of total protein was used for sodium dodecyl sulfatepolyacrylamide gel electrophoresis and transferred onto polyvinylidene difluoride membranes (Millipore, USA). The membrane was incubated with primary antibodies against GLUT2 (1:1000, Affinity Bioreagents, USA), MYC (1:1000, Affinity Bioreagents, USA), and GAPDH (1:5000, Affinity Bioreagents, USA), followed by the secondary antibody (1:5000, Affinity Bioreagents, USA). The blots were detected using an enhanced chemiluminescence detection kit (Millipore, USA). The expression analysis was performed using the ImageJ software. The ratio of densitometry value to the corresponding GAPDH was used to reflect the relative protein expression.

## Results

### ***C. albicans*** Affected the RNA Expression Profile in HUVEC Cell Lines

To clarify the effects of *C. albicans* on endothelial cells, we downloaded the GEO database of GSE7355 from the GEO database. By calculating residuals.sign, residuals, weight, RLE, NUSE, and RNA degradation, the data quality was acceptable (Figure S1A–F). The heat map analysis result showed a quite different expressed gene profile between the control HUVEC monolayer and *C. albicans*-infected HUVEC monolayer (Figure S2). The volcano plot further described a difference in gene expression profile between the two groups. For instance, many genes were considerably upregulated and labeled in red color, whereas some genes were remarkably downregulated and labeled in blue color (Figure S3).

### ***C. albicans*** Infection Affected Apoptosis, Metabolism, and Inflammation Related Pathways

**Fig. 1:**
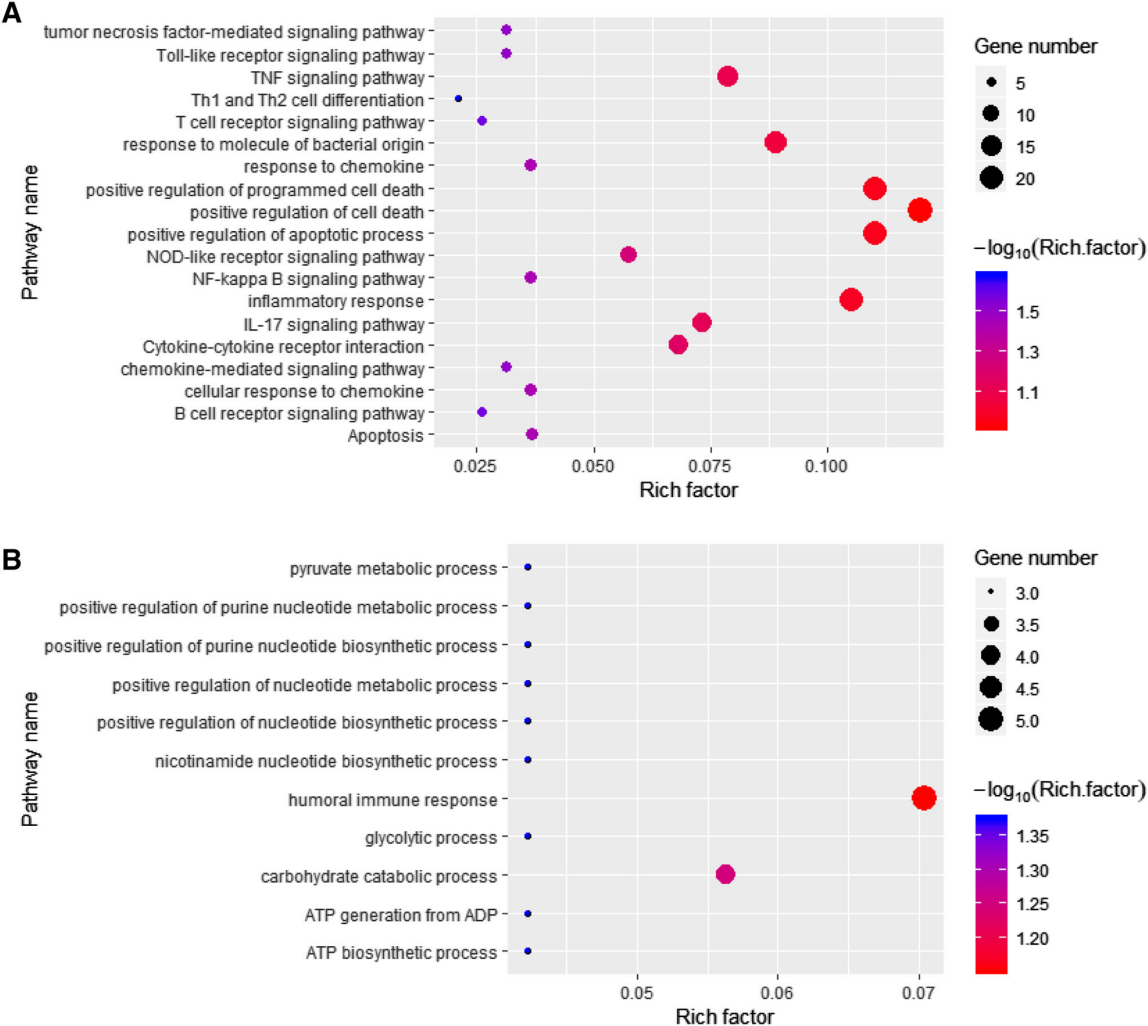
Effects of *Candida albicans* infection on biological processes and pathways in HUVEC cells. **a** Pathway analysis of the upregulated genes by *C. albicans* infection revealed an activation of immune and inflammation related pathways including TNF, Toll-like receptor, T cell receptor, NF-kappa B, and chemokine-mediated signaling pathway. **b** Pathway analysis of the downregulated genes by *C. albicans* infection revealed an inhibition on metabolism related processes like synthesis of nicotinamide nucleotide and ATP (false discovery rate < 0.05, |log2fold change| ≥ 0.6)

**Fig. 2:**
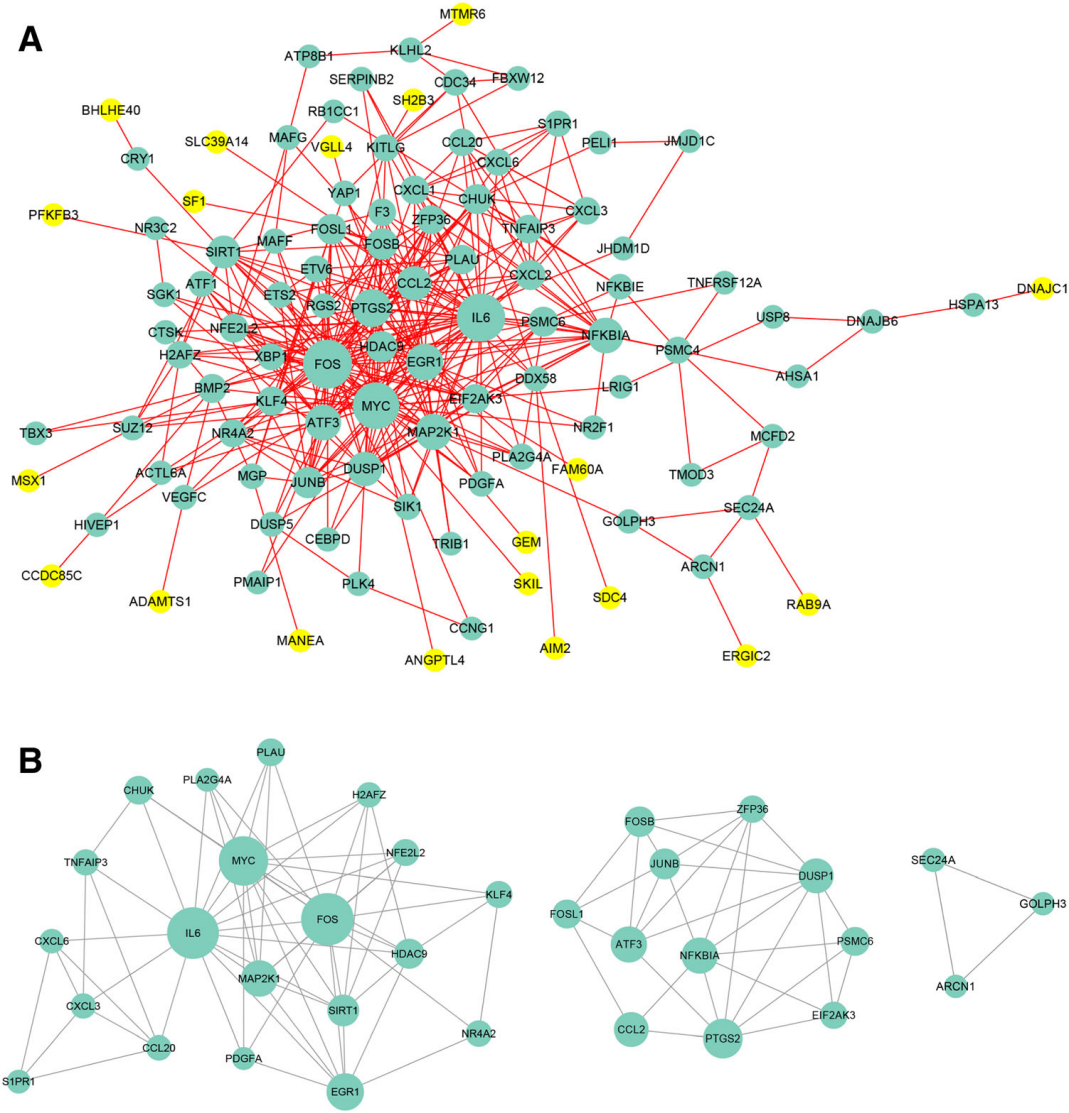
PPI network of DEGs. **a** PPI analysis of the upregulated genes by *Candida albicans* infection by Cytoscape software, where the node size reflected the gene interaction degree, larger circles indicated higher gene interaction degree. **b** Seed nodes analysis of the PPI network by MCODE. Three modules identified by MCODE showed the candidate targets of treating *C. albicans* infection, with the network scoring of 6.737, 6, and 3.

The omics analysis was then conducted with the screened DEGs. The GSEA analysis results showed that *C. albicans* infection could affect the apoptosis and oxidative phosphorylation processes. Furthermore, the *C. albicans* infection could affect the inflammatory response and its related signaling pathways, namely, NFKB, IL6-JAK-STAT3, and IL2-STAT5 pathways (Figure S4). Further analysis of the DEGs revealed that *C. albicans* infection extensively promoted immune- and inflammation-related signaling pathways, such as TNF, Toll-like receptor, T cell receptor, NF-kappa B, and chemokine-mediated signaling pathway (Fig. 1a). The metabolism-related pathways, such as glycolytic and carbohydrate catabolic processes, were almost completely suppressed by *C. albicans* infection, thereby inhibiting synthesis of nicotinamide nucleotide and ATP (Fig. 1b). Multiple biological processes and pathways were implicated in HUVEC monolayer by *C. albicans* infection. On this basis, we constructed *C. albicans*-related network using PPI information of DEGs (Supplemental table). This network consisted of 105 nodes and 360 edges (Fig. 2a). By using the CentiScape and MCODE APPs, we identified the topological network with features of Degree and K-Core. Three hub networks were screened with the network scoring of 6.737, 6, and 3, respectively (Fig. 2b). As a result, some potential hub nodes, such as MYC, IL6, FOS, and NFKBIA, were identified as candidate targets for treating *C. albicans* infection (Table 1).

**Table 1:** Top hub proteins in the PPI network based on interaction degree

Gene name	Degree unDir	Bridging unDir	Eigen vector unDir	Radiality unDir	Stress unDir
IL6	47	13.838	0.334	6.683	15,962
FOS	43	9.326	0.315	6.602	11,290
MYC	40	13.342	0.282	6.621	14,748
PTGS2	27	15.575	0.248	6.491	5250
MAP2K1	24	12.665	0.197	6.385	5532
EGR1	23	10.907	0.215	6.416	3646
CCL2	23	12.011	0.201	6.329	3150
NFKBIA	21	38.857	0.195	6.422	7938
ATF3	21	6.814	0.185	6.298	1976
DUSP1	19	17.998	0.184	6.323	2984

### Candidate Drugs Screening for Treating ***C. albicans*** Infection

**Fig. 3:**
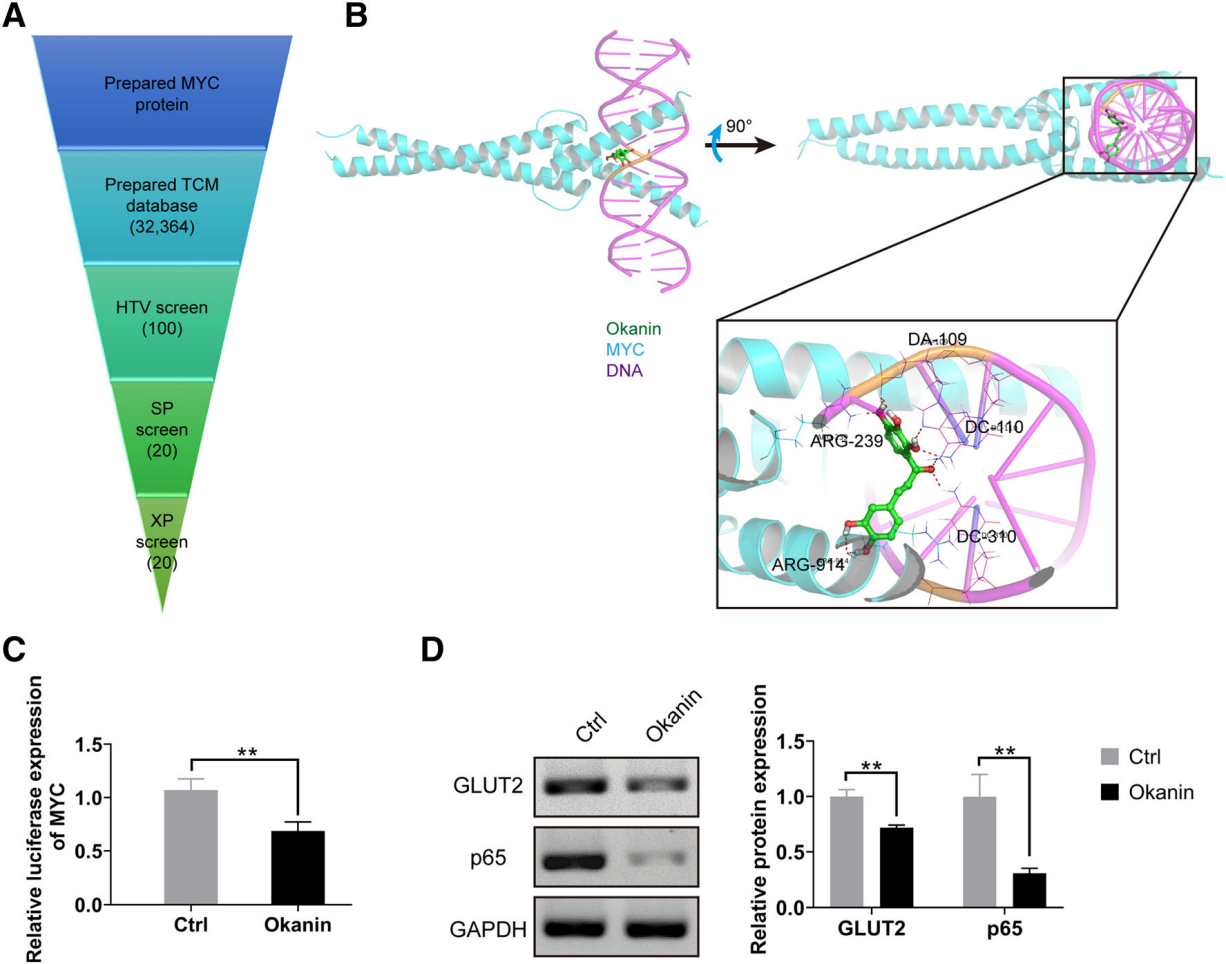
Receptor–ligand interactions of compound. **a** Protocol flowchart of MYC inhibitor discovery strategy, ten candidate inhibitors including okanin were screened at last through HTV, SP, and XP screen methods. **b** Binding model of MYC–DNA complexes with okanin (PDB code: 1NKP) through molecular docking method. The results showed that okanin interacted with two key amino acid residues in the DNA binding site of MYC. **c** Okanin could significantly inhibit the transcriptional regulatory activity of MYC by luciferase assay. **d** Okanin significantly inhibited the expressions of GLUT2 and p65, which were the downstream proteins of MYC (n = 3, **P < 0.01)

In accordance with the omics analysis, MYC was detected to be a hub node and an important gene related to inflammation and transformation of inflammatory cancer. Therefore, we chose the gene MYC for drug screening. A systematic strategy for identifying TCM molecules were designed by using structure-based VS. The high-throughput virtual screening process is shown in Fig. 3a. HTV screen method yielded 100 TCM molecules with the highest MYC score. Furthermore, twenty TCM molecules were further screened out form the previous 100 TCM molecules. Finally, 10 TCM molecules were obtained (Table 2). We performed extra precision calculation with MYC–DNA complex. Among these molecules, okanin had the highest docking score. The interactions between MYC–DNA and okanin are shown in Fig. 3b. Okanin interacted with key amino acid residues (Arg-239 and Arg-914) in the DNA binding site of MYC via two hydrogen bonding interactions. In DNA helix, okanin formed hydrogen bond interaction with three bases. Okanin could inhibit the binding sites of MYC and DNA, thereby inhibiting the transcriptional activity of MYC. To further demonstrate the activity of okanin on treatment of C. albicans infection, the luciferase assay of MYC and MYC downstream protein expression of GLUT2 and p65 were conducted [21, 22]. The luciferase assay results showed that okanin could significantly inhibit the transcriptional regulatory activity of MYC (Fig. 3c). Meanwhile, the expressions of MYC downstream proteins of GLUT2 and p65 were also significantly inhibited by okanin (Fig. 3d).

**Table 2:** Detailed docking score of top ten screened TCMs

Compound name	Docking score	Glide evdw	Glide ecoul	Glide energy	Glide einternal	Glide emodel
Okanin	− 9.97	2.14	− 30.85	− 28.71	2.52	− 46.83
Prolithospermic acid	− 9.38	− 25.15	− 24.53	− 49.67	7.15	− 50.26
Eximine	− 8.91	− 24.27	− 18	− 42.27	12.27	− 60.42
Chalconaringenin	− 8.32	− 18.09	− 21.36	− 39.44	3.33	− 48.89
Maclurin	− 8.27	− 21.93	− 20.63	− 42.57	2.57	− 56.93
(+)-Catechin	− 6.93	− 23.54	− 12.38	− 35.92	2.23	− 48.89
Licochalcone B	− 6.93	− 15.63	− 14.66	− 30.29	7.53	− 23.07
Pinobaksin	− 6.63	− 20.57	− 7.66	− 28.23	0.14	− 44.29
Citreorosein	− 6.48	− 26.89	− 21.76	− 48.65	1.95	− 63.36
2'-Methoxy-3,4,4'-trihydroxychalcone	− 6.35	− 20.75	− 17.07	− 37.82	6.79	− 49.1

## Discussion

Microorganisms in the lumen enter the parenchymal tissue through their interaction with endothelium and then cause infection by increasing the vascular permeability [23]. This occurrence is the first and key step of disseminated infections [24]. Hence, vascular endothelial cells play a key role in the early stage of hematogenous disseminated infections and is a tool for studying fungal infections [25]. Thus, studying the effect of *C. albicans* on vascular endothelial cells can clarify the pathogenesis of disseminated candidiasis. Although there have be some treatments for *C. albicans *[26], more effective treatment methods need to be selected and developed. *C. albicans* invades human host cells and is associated with cell motility, cytoskeleton actin, PI3K signaling pathway, and membrane receptors EGFR and Erbb2 [27–30]. From a microbial point of view, the activity of pathogenic fungi, mycelial growth, and the virulence factors of *C. albicans*, such as als3 and Ssa1, all play an important role in the whole process [31].

Hub genes play vital roles during the progression of *C. albicans* infection. Although many studies on *C. albicans*-infected endothelial cells are available, much effort is still needed to identify hub genes and develop candidate drugs that may inhibit *C. albicans* infection. Pathway enrichment analysis indicated that TNF signaling pathway, T cell receptor signaling pathway, response to chemokine, positive regulation of apoptotic process, and inflammatory response pathways were overrepresented among the upregulated genes.

The PPI network was constructed with 162 nodes/DEGs. The PPI network and the topological index revealed that IL6, FOS, and MYC were the most important in this PPI network. These proteins have a high degree of expression and a central regulatory role in the PPI network. Hence, they are likely to be regulatory hubs.

MYC, a transcription factor, can bind DNA in a non-specific manner [32]. MYC gene is an important member of MYC gene family. It is not only a translocation gene but also an adjustable gene regulated by many substances. It can make cells proliferate indefinitely and obtain the function of immortalization; it promotes cell division [33, 34]. It is also involved in cell apoptosis and is related to the development of various tumors [35]. In this study, we used the crystal structure of MYC–DNA complex to screen TCM molecules that may inhibit MYC activity. By multi-level virtual screening, 10 TCM molecules with potential inhibitory activity were obtained; among these molecules, okanin had the highest docking score.

This study demonstrated that okanin, a major effective constituent of *Bidens* (Asteraceae), may inhibit *C. albicans* infection by inhibiting MYC. Okanin can lower the mRNA and protein level of IL6 in the LPS-induced pro-inflammatory model [10]. It also potently inhibited a number of pro-inflammatory responses in cells [8]. The structure of okanin contained α-β unsaturated carbonyl moiety that was composed of two hydroxyphenyl rings and a three-carbon unit. The binding model of okanin and MYC–DNA complex revealed that okanin was in the middle of MYC binding to DNA and inhibited the transcriptional activity of MYC. Okanin interacted with Arg239 and Arg914 of MYC to form two hydrogen bonds. Okanin also interacted with DA109, DC110, and DC310 on DNA [18]. Therefore, blocking interaction of MYC and DNA was observed. Okanin inhibited MYC transcriptional activity, possibly also inhibiting the transformation of inflammatory cancer caused by *C. albicans* infection.

## Electronic Supplementary Material

Below is the link to the electronic supplementary material

Supplementary Material 1

Supplementary Material 2
