# Supplementary material for: Identification of Key Candidate Genes and Pathways of Candida albicans-Infected Human Umbilical Vein Endothelial Cells and Drug Screening
Source: Indian J Microbiol. 2019 Dec 13;60(1):62–9. doi: 10.1007/s12088-019-00847-5 (PMC7000633; doi:10.1007/s12088-019-00847-5)
Supplement: Supplementary file 1 — Supplementary Material 1 [file 12088_2019_847_MOESM1_ESM.docx]

**Supplemental Materials**


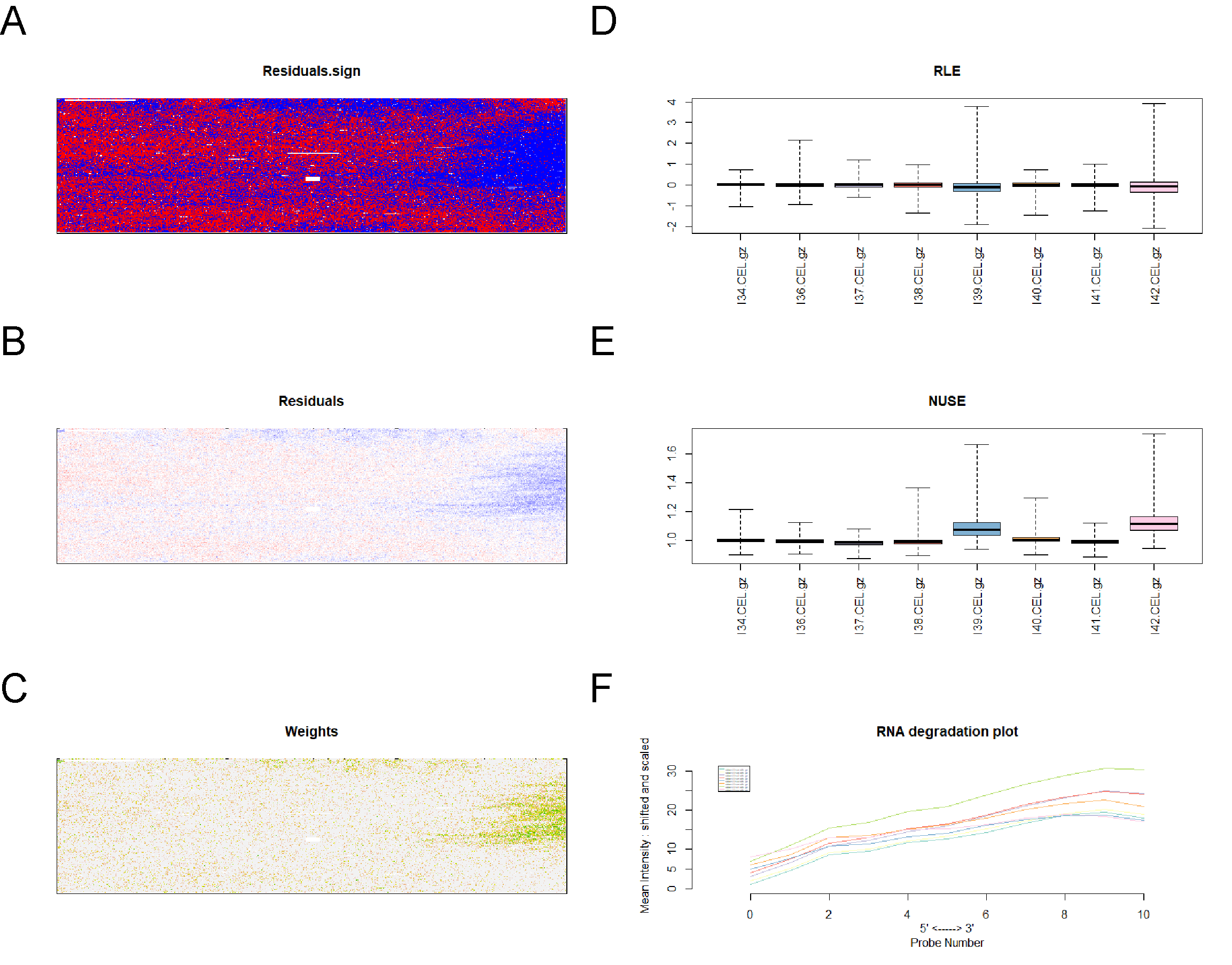


**Figure S1. Data quality analysis of GSE7355.** Calculations of **A.** Residuals.sign, **B.** Residuals, **C.** Weight, **D.** RLE, **E.** NUSE, and **F.** RNA degradation demonstrated that the data quality of RNA expression profile was acceptable.


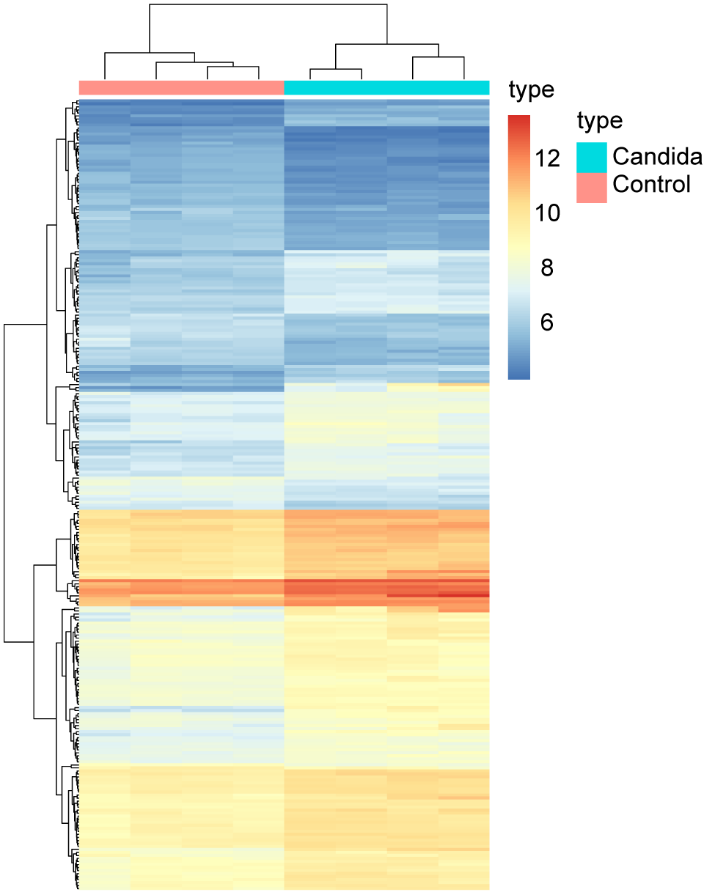


**Figure S2. Gene expression profiling analysis.** Heatmap analysis for comparison of gene expression profiling between untreated HUVEC monolayer and Candida albicans-infected HUVEC monolayer.


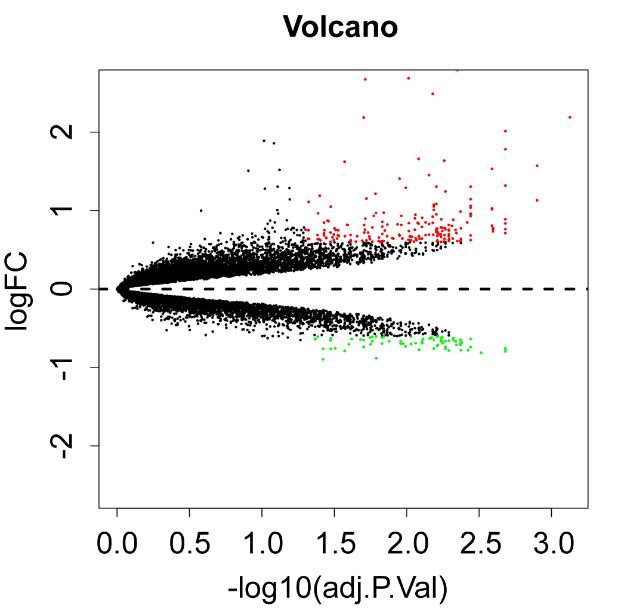


**Figure S3. Gene expression profiling analysis.** Volcano plot for the DEGs with the *C. albicans* infection, where red pots meant upregulated genes and green pots meant downregulated genes. (False discovery rate < 0.05, |log2fold change| ≥ 0.6).


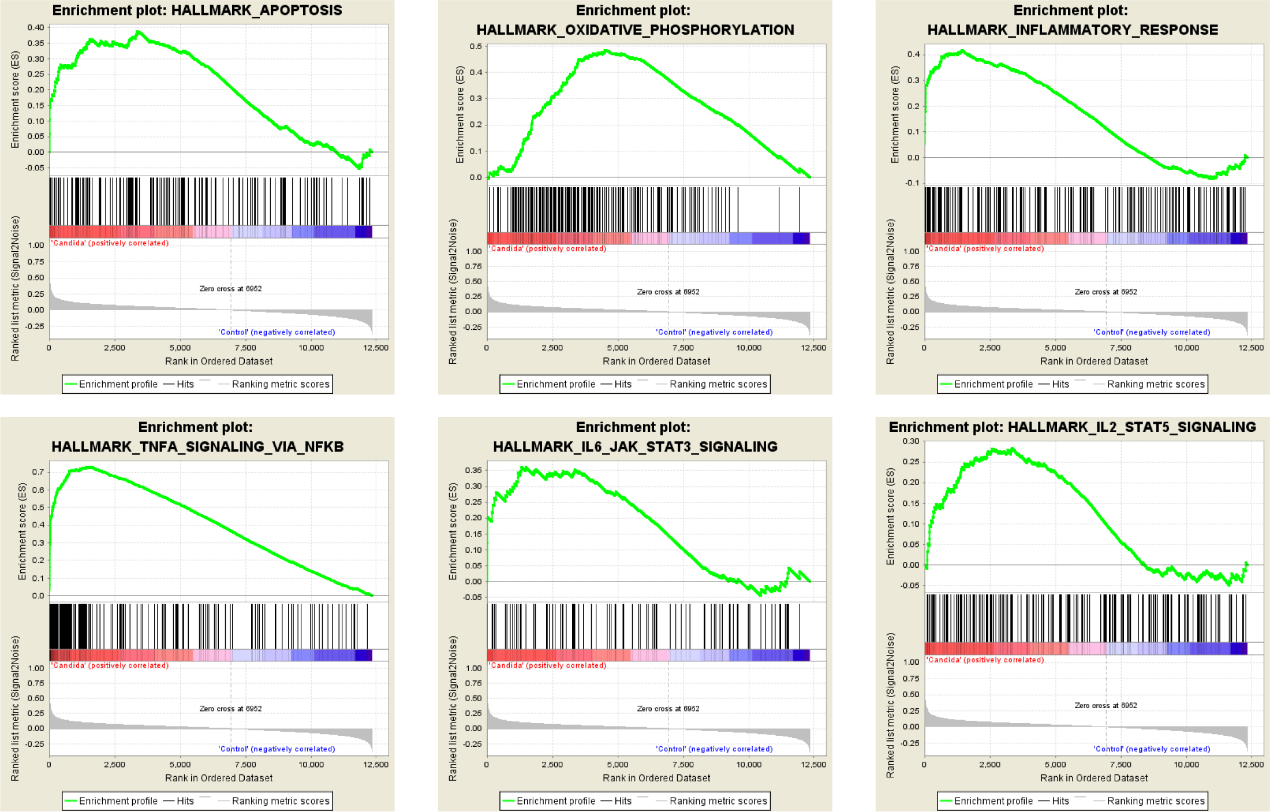


**Figure S4.** Gene expression profile analysis by GSEA revealed an upregulation of apoptosis, oxidative phosphorylation, and inflammatory response processes; the signaling pathways of NFKB, IL6-JAK-STAT3, and IL2-STAT5 were also activated by C. albicans infection.
